# Three Risks That Caution Against a Premature Implementation of Artificial Moral Agents for Practical and Economical Use

**DOI:** 10.1007/s11948-021-00283-z

**Published:** 2021-01-26

**Authors:** Christian Herzog

**Affiliations:** Ethical Innovation Hub, Institute for Electrical Engineering in Medicine, University of Lübeck, Ratzeburger Allee 160, 23562 Lübeck, Germany

**Keywords:** Machine ethics, Artificial moral agents, Artificial intelligence, Robot ethics, AI ethics

## Abstract

In the present article, I will advocate caution against developing artificial moral agents (AMAs) based on the notion that the utilization of preliminary forms of AMAs will potentially negatively feed back on the human social system and on human moral thought itself and its value—e.g., by reinforcing social inequalities, diminishing the breadth of employed ethical arguments and the value of character. While scientific investigations into AMAs pose no direct significant threat, I will argue against their premature utilization for practical and economical use. I will base my arguments on two thought experiments. The first thought experiment deals with the potential to generate a replica of an individual’s moral stances with the purpose to increase, what I term, ’moral efficiency’. Hence, as a first risk, an unregulated utilization of premature AMAs in a neoliberal capitalist system is likely to disadvantage those who cannot afford ’moral replicas’ and further reinforce social inequalities. The second thought experiment deals with the idea of a ’moral calculator’. As a second risk, I will argue that, even as a device equally accessible to all and aimed at augmenting human moral deliberation, ’moral calculators’ as preliminary forms of AMAs are likely to diminish the breadth and depth of concepts employed in moral arguments. Again, I base this claim on the idea that the current most dominant economic system rewards increases in productivity. However, increases in efficiency will mostly stem from relying on the outputs of ’moral calculators’ without further scrutiny. Premature AMAs will cover only a limited scope of moral argumentation and, hence, over-reliance on them will narrow human moral thought. In addition and as the third risk, I will argue that an increased disregard of the interior of a moral agent may ensue—a trend that can already be observed in the literature.

## Introduction

In the present article, I will advocate caution against the utilization of preliminary forms of artificial moral agents (AMAs) based on the notion that their implementation will potentially negatively feed back on the human social system and on human moral thought itself and its value—e.g., by diminishing the breadth of employed ethical arguments and the value of character. I will indicate potential ways in which intermediate steps in the development and deployment of AMAs will likely be a driver of social inequality and a narrowing of human moral thought. I base my claims on the observation that the current economic system is rewarding increases in productivity or efficiency, which—when applied to the moral domain—may result in adverse effects.

My argument will proceed from two interrelated perspectives: First, I will argue that already the utilization of premature forms of AMAs may consolidate and amplify power asymmetries by allowing the already powerful to disproportionately increase their power by increasing their ’moral efficiency,’ i.e., their ability to decide on a larger amount of moral issues per period. I will sketch the effects of such ’moral replicas’ as a thought experiment to illustrate and support my claims about the likelihood of such scenarios by referencing actual progress made towards them.

Second, even if the likely perspective of increased power asymmetries and social disparities can be thwarted, e.g., by political means, I suggest that the pursuit of AMAs may yield another severe effect: The perpetuated and widespread—i.e., even egalitarian—use of preliminary forms of AMAs may be likely to diminish the breadth and depth of concepts employed in moral arguments and a devaluing of the processes interior to moral agents. To show this, I will argue that augmenting or even outsourcing moral decision-making via AMAs under the dogma of increasing our (moral) efficiency will gradually influence our formation of moral intuition and, consequently, expertise, cf. (Pizarro and Bloom 2003; Sauer 2012). When increasingly relying on these systems, the breadth of our moral thought may match—or rather may be constrained by—that of the AMAs. I will base my argument on a second thought experiment that considers the mistaken notion that automating moral deliberation can be likened to automating computations using calculators, cf. (Cave et al. 2018).

By premature forms of AMAs, I regard devices that are capable of moral evaluations that could be taken as decisions or decision support on which people might act. Such systems are already on the horizon. Take, e.g., the recently released natural language processing system GPT-3, which is capable of producing text output that is barely distinguishable from human outputs (Weinberg 2020). It is conceivable that such a system may produce outputs that could be taken as, e.g., management directives. Other examples include the so-called Moral Choice Machine (Schramowski et al. 2020), which finds clues of human moral norms embedded in texts and is capable of answering to simple yes-or-no-questions in response to moral questions. Premature AMAs in this sense, hence, might not yet be awarded rights based on their moral status, but may autonomously take action, e.g., by being trained to follow the moral standards of a group, or even a single person’s.

Because of an economic system that does not support justice and fairness as its primary goal, AMAs may not even attain a level of sophistication comparable to the current best understanding of human morals due to a lack of incentives. To be quite clear: I will not reiterate the threat of moral deskilling as such, cf. (Vallor 2015). Instead, my argument hinges upon the notion that any reliance on AMAs that incorporate only a premature and, hence, limited understanding of human morality will perpetuate this limitation, reducing diversity in the very thing AMAs try to augment or improve. These effects may even carry over to scientific investigations, e.g., exemplified by what can already be observed from a common trend in machine ethics to limit the scope of moral agency by disregarding for what is interior to a moral agent, see, e.g. (Floridi and Sanders 2004; Beavers 2011).

However, I will argue that the scientific development of AMAs aiming at better understanding human morality poses no real threat, as long as scientific rigor includes analysis and documentation of the limits of the findings inherent to the computational methods. Rather, I argue against a premature practical utilization of the scientific results.

Hence, this article’s arguments primarily focus on what may get lost within a notion of ’morality’ by utilizing AMAs prematurely. To the best of my knowledge, this perspective has not been fully explored in the literature. Instead, arguments in favor rather focus on the necessities for, motivations of, and promises offered by developing AMAs, see, e.g. (Gips 1991; Moor 2006; Anderson 2011; Cave et al. 2018). In turn, arguments against their creation either rely on refuting claims made by proponents (van Wynsberghe and Robbins 2019), arguing for the immorality of constructing AMAs of particular types, see, e.g., (Tonkens 2009), or cautioning what their creation might imply in terms of their moral status, e.g., (Bryson 2018). Only a few works highlight aspects in which humankind is prone to a loss due to AMAs. These include risks of moral deskilling (Vallor 2015) or of devaluing the relevance of the interior (e.g., intentions) of moral agents (Beavers 2012). However, none of these works focus on the complexities of developing AMAs in the broader context that is the reality of human society. Human society’s intricate social, political, and economic systems make linear progress towards AMAs that fulfill the positive prospects of machine ethics rather unlikely.

In pursuing and elaborating on my arguments outlined above, I intend to extend these lines of thought while staying charitable to the proclaimed objectives of machine ethics to help understand and perhaps improve human moral deliberation. Elaborating on risks always remains a speculative endeavor, but so does the promise of achieving scientific success. I do not intend to assume an overly conservative stance. Instead, this article is supposed to act as an invitation to proponents of AMAs to consider how proclaimed goals of increased social equality and improved human morality are to be realized. First, however, a brief overview of different notions of artificial moral agency is given.

## The Debate on Artificial Moral Agents in Machine Ethics

Moor (2006), coined a now almost canonical taxonomy of artificial moral agency: Artificial ’ethical-impact agents’ do not exhibit self-directed action, but still have ethical impacts on the environment. ’Implicit ethical agents’ are constrained in their actions to comply with moral standards, while ’explicit ethical agents’ can justify their largely autonomous actions. ’Full ethical agents’ are denoted to possess human-like capacities, such as free will and self-consciousness. This has received criticism of different kinds. Beavers (2011), rejects it as anthropocentric and offers a distinction between a ’moral agent’ as any agent that “does the right thing morally, however determined” (Beavers 2011, p. 5) and a ’responsible moral agent’ that is fully responsible and accountable. Cave et al. (2018), criticize Moor’s various qualitative uses of the word ’ethical’ and instead offer the notions of ’ethically aligned machines’ and ’machines capable of ethical reasoning.’ Ethically aligned machines act following “the interests and values of the relevant stakeholders in a given context” (Cave et al. 2018, p. 563), while machines capable of ethical reasoning can run “processes that are concerned with solving ethical problems” (Cave et al. 2018, p. 564). Remarkably, despite claiming to cover the motivations and risks of the entire field of machine ethics, Cave et al., however, drop regard for the demand that “ethical reasoning requires an understanding of the significance of the ethical issues at stake” (Cave et al. 2018, p. 564) and therefore, in their simplified taxonomy, stop short of differentiating between Moor’s notion of explicit and full ethical agents.

Hence, both Beavers and Cave et al. appear not to be interested in incorporating any relevance of the moral interior of an agent into their taxonomies. Within their approaches, only the effects of an agent’s actions or any process that can arrive at a solution are deemed relevant, but not the qualities of the process involved. A narrowing of a taxonomy of moral agency seems unfortunate. It is perhaps a result of machine ethics’ focus on what concepts may be grasped within the confined realm of implementation, cf. (Tonkens 2009). Such and similar constrictions of conceptions of moral deliberation are at the heart of my criticism of pursuing AMAs.

However, my main argument, why these restricted conceptions may ultimately prevail, rests on the practical and economic constraints under which AMAs will be introduced to society. This will lead to AMAs being mostly aimed at increasing productivity and efficiency rather than being aimed at improving human moral deliberation or reducing social inequality, which constitute the proclaimed goals of machine ethicists, cf. (Anderson 2011).

In what follows, however, I will first provide a brief overview of the debate on whether humankind should pursue AMAs in principle. In a rebuttal, van Wynsberghe and Robbins (2019) first survey often-stated reasons for pursuing the development of AMAs. They list claims that AMAs...

(i) ...are inevitable (Wallach and Allen 2011),
(ii) ...will be required for preventing human harm (Scheutz 2016) and achieving ethical alignment of autonomous systems (Tonkens 2009),
(iii) ...will be required in unpredictable environments, e.g., (Allen et al. 2006),
(iv) ...will increase trust in autonomous systems, e.g., (Wiegel 2006),
(v) ...can prevent being exploitation for malicious purposes,
(vi) ...may be superior moral reasoners, e.g., (Gips 1991), and
(vii) ...will help better understand human morality, e.g., (Moor 2006).

In brief, the rebuttals consist of arguing that (i) machines need not be delegated a moral role, but virtually every machine is an ’ethical-impact agent’ (cf. Moor (2006), and the explanation above), not all of which can or should be developed into AMAs, (ii) ethics should not be conflated with safety, while the safe design of technological systems is always a viable solution, (iii) restricting complexity is a superior and well-established procedure for handling unpredictability, also with humans, (iv) intentionality is limited to beings and, hence, while technical systems need to be relied upon, trust in them is misplaced, cf. (Simon 2010) and (v) the full extent and potential of misuse of technology is not even understood for ’ethical-impact agents’. I will not argue against the rebuttals to claims (i) to (v). Here, I regard claims (vi) and (vii) as central as they arguably seem to be the noblest endeavors of machine ethics that would constitute a true epistemological advancement. and van Wynsberghe’s and Robbins’ rebuttals to them as unsatisfactory.

I deem both of van Wynsberghe and Robbins’ rebuttals to claims (vi) and (vii) as unsatisfactory. This is because, for (vi) and (vii) van Wynsberghe and Robbins (2019) draw on error theory, i.e. the notion that there are no objective moral truths, and moral skepticism, i.e., the notion that humans cannot know objective moral truths, as relevant but debatable theories that, however, do not conclusively rule out that the pursuit of AMAs will lead to humankind understanding its morality better and that this may still lead to superior moral reasoning, regardless of whether it needs to be augmented by machines or not. In contrast, I believe that the notion of AMAs for personal use shows that we should take risks of irretrievable and adverse change seriously—and I will argue that this may happen by society adopting scientific advancements in AMAs prematurely.

My argument does not rely on an exact definition of ’mature.’ Instead, the notion of premature AMAs in the present article lives in the grey area between ’implicit ethical agents’ and ’explicit ethical agents’. I will consider two thought experiments in the sequel. In the first, I consider ’moral replicas’ that simply adhere to the moral norms of a particular individual and produce corresponding outputs that are not necessarily accompanied by justifications. In the second, I consider ’moral calculators’ capable of presenting justifications, but typically incapable of acting autonomously.

I will argue that both thought experiments show that within our current economic system, which values productivity increases above all else, premature forms of AMAs will fail to realize the laudable goals presented in the machine ethics literature. In brief, they may (i) reinforce social inequalities, (ii) narrow human moral thought, and (iii) contribute to a growing disregard of an ethical agent’s moral interior. Above, I have already hinted at works that appear as purveyors of such disregard.

## Moral Replicas: A First Thought Experiment

In this section, I propose a thought experiment that highlights how preliminary forms of AMAs are likely to lead to a consolidation and even amplification of existing power asymmetries. Scientific research towards AMAs, originally aimed at investigating human morality, might spawn economically motivated spin-offs that dispose of the original scientific goal when having matured to be amenable to a business case. As an example, consider a service provider specialized in tailoring advanced personal assistants to their customers’ needs. Such a company may be approached by powerful individuals, such as CEOs of successful enterprises, who aim at acquiring virtual assistants that can manage branches of their businesses. It stands to reason that such a CEO aims at running her or his enterprise according to personal moral convictions. Such a scenario may avoid discussions about the accuracy of moral frameworks, the existence of objective moral truth, or even the question about whether an AMA should be able to act in a morally wrong way as a requirement for true responsible agency, cf. (Beavers 2011, p. 8). Instead, it is a perfectly conceivable application that may be facilitated by training-based AI research in machine ethics, cf. (Schramowski et al. 2020). Instead of identifying human biases, cf. (Caliskan et al. 2017), such approaches try to replicate human biases towards certain societal norms. As such, approaches capable of extracting implict moral stances from data aim to avoid an issue raised by Gordon (2020), namely that insufficient ethical expertise within a development team gives rise to errors in designing algorithms for moral evaluation.

Systems such as Google Duplex are already able to perform rather menial tasks, such as making appointments (Chen and Metz 2019). It is conceivable that soon such assistants are also capable of performing tasks that involve making a wide range of decisions. The utility of such assistants appears obvious: The CEO would not be interested in improving humankind’s morality, but rather in, what I call, increasing her or his ’moral efficiency,’ i.e., increasing the number of moral decisions taken aligned with a personal moral code during a specific period.

Regulations may demand that moral replicas be offered only aligned to some set of democratically approved moral norms. However, in practice, it might be challenging to determine when decisions of such personal assistants touch on morally salient aspects. A company offering advanced personal assistants might face economic pressure in making covert concessions to the client’s demand for a true moral replica, instead of delivering a personal assistant replicating the client’s moral stances only as long as they align with overall moral norms.

I will elaborate on the associated risk that AMAs may reinforce social inequality in Sect. 5.1.

## Moral Calculators: A Second Thought Experiment

Consider another byproduct of the scientific occupation with human morality through building AMAs as a device aimed at supporting the moral deliberation process of humans, e.g., by posing relevant questions about a particularly morally salient situation. Such (preliminary) forms of AMAs have been suggested in the literature as so-called ’Socratic assistants’ (Lara and Deckers 2019), ’moral calculators’ (Cave et al. 2018), or ’artificial moral advisors’ (Giubilini and Savulescu 2018) that may significantly speed up and correct the human moral deliberation process just like actual calculators do with mental arithmetic. After all, a meta-survey highlights the potential of special instruction concerning calculators and cites evidence of longer-term effects indicating that students “were more successful in mental calculation; and that they were better able to tackle real world problems.” (Ruthven 1996).

Now, if a calculator were a fair analogy, this suggests that we need not worry about the use of technological aids for moral deliberation. Rather, ’moral calculators’ would likely be assumed to allow for a better grasp of higher-level moral concepts, speed up our deliberation, and reduce oversights. I acknowledge that this may well be true—but only if we could implement the correct way to assist in moral deliberation right from the start. Humankind may, at some point, find this one correct moral framework, but currently, this is almost certainly not so. In other words: As technology progresses and engineering ingenuity reigns lose on the marketplace, any prospective product of a ’moral calculator’ is bound to be biased. I will elaborate on this risk in Sect. 5.2.

## Risks of Prematurely Implementing Artificial Moral Agents

In the following, I will elucidate on three possible risks that caution against a premature implementation of AMAs. I will draw on the thought experiments of the moral replica and the ’moral calculator’ as illustrations that premature AMAs are likely to (i) reinforce social inequalities, (ii) lead to a narrowing of human moral thought, and (iii) lead scholars and citizens to further disregard an agent’s moral interior in referring to moral agency. I will consider each risk in turn.

### Reinforcement of Social Inequalities

The thought experiment of ’moral replicas’ is intended to showcase how premature forms of AMAs might give rise to a potentially highly successful business case. Besides potential qualitative advances, many AI ventures promise increased productivity through automation. Increasing ’moral efficiency’ via ’moral replicas’ would not only constitute an increase in power, it would further also potentially pay off well. Of course, the CEO might as well employ humans to act in her interests. Software, however, could provide a cheaper solution. A human, in turn, might also be interested in his or her own projects, e.g., such as saving the environment, but with ’moral replicas,’ any corrective action against the leadership’s will from lower management tiers will be rendered impossible. At possibly a fraction of the cost of human personnel, this presents the ultimate and unchallenged power to lead. Apart from ’moral replicas’ in the business world, it is easy to conceive of other use-cases, where positions of power can be rendered unchallenged by morality-replicating AMAs, e.g., politics. However, I propose that the conflation of mechanisms for economic success and an unregulated approach to exploiting preliminary results from machine ethics holds the most severe risk of reinforcing social inequalities.

Evidence suggests that employing AMAs as ’moral replicas’ in management is not unlikely. Newspaper articles already discuss the prospect of replacing management by AI, cf., e.g., (Novita 2019), and cite surveys that indicate a significant margin of people in favor of such practice, or list potential advantages, such as that robot managers will act “objectively, professionally, 24/7” (Kruse 2018). A study by Oracle even claims that half of the workers surveyed have already taken automated managerial advice (He et al. 2019). However, what is naïvely framed as the end of workplace disputes, cf. (Kruse 2018), AI technology capable of managing is possibly not only likely to incorporate biases—it is designed to be so, especially if intended as a ’moral replica,’ cf. (Schramowski et al. 2020). Premature utilization of data-based algorithms has already sparked a healthy debate about the issue of algorithmic bias, i.e., the tendency to reinforce rather than to alleviate human biases, discrimination, and racism, see, e.g., (Koene 2017; Angwin et al. 2016). Even in the presence of regulation demanding objectivity, there are considerable incentives for the provider of the personal assistant to favor the CEO’s personal moral stances in the programming of the AMA. Such replications of biases would not be easy to detect and—at the very least—one would not expect the ’moral replica’ to truly challenge to the CEO.

However, it is imaginable that society finds a way also to endow the less privileged with the benefits of morality-replicating AMAs. For that purpose, I will turn to outline a potentially even graver consequence of the premature use of preliminary forms of AMAs, which may confine our conception of ’morality’ itself.

Having considered scenarios in which premature forms of AMAs are employed by the privileged to consolidate their power, I turn to maintain that, in addition, a lock-in situation might ensue, in which the premature and widespread use of AMAs has perpetuated reductionist notions of human moral thought and moral frameworks. Then, science seeking to understand and improve human morality may only find what the use of technology has reduced in richness. Thus, I maintain that AMAs—even if available to everyone in an almost egalitarian way—could alter our understanding and ability of moral deliberation to a detrimental effect.

To begin with, also morality-replicating AMAs will likely not advance humankind’s understanding of ethics and how human moral deliberation works. All that matters would be the power to copy one’s moral compass as accurately as possible. From an outside perspective, decisions taken by such an AMA may not even be discernible from the original human being. As discussed above, this may not worry some authors that focus on “mindless morality” to avoid questions of “mental states, feelings, emotions and so on” (Floridi and Sanders 2004). But would morality-replicating AMAs really lead to a narrowing of humankind’s conception of morality as a whole?

### Narrowing of Moral Thought

The analogy of an arithmetic calculator and a ’moral calculator,’ as introduced in Sect. 4, may be appealing at first. However, it is severely lacking. Computing numbers is based on a human-defined axiomatic system. If at a loss, anybody can look up the rules of basic arithmetic and retrace relevant steps for any computation manually or by alternative tools. Furthermore, a calculator entirely relies on human input. ’Artificial moral advisors,’ as proposed by Giubilini and Savulescu (2018), would aid in collecting information in humanly impossible ways “to the extent that such information can be made available in a way that can be modeled and used by software” (Giubilini and Savulescu 2018, p. 174). Not only will the information not be exhaustive (neither can humans collect all relevant information), it will also be limited by what can be accessed by sensors and forms of data acquisition. Also, when will humans have the leisure to check whether the information considered was also relevant? Automation bias is likely to eventuate, which is already an issue discussed, e.g., in the context of clinical decision-support (Goddard et al. 2011).

Systematic errors and biases in ’moral calculators,’ however, would be much more difficult to detect because people disagree about morals, and it is not clear against which standard they are to be evaluated. This is also true considering the potential alignment of ’moral replicas’ with society’s moral norms. In due course, their use will have skewed decisions potentially unfairly with little to no ways available to detect and correct the wrongs. However, I am not making my point based on doubts that there, in fact, exists an objective moral truth, such a ’moral calculator’ could implement. This has been discussed elsewhere, see (van Wynsberghe and Robbins 2019). After all, one may rightfully object that also human moral councilors may be biased, incurring moral wrongs. My point is rather that, while humankind is still in pursuit of an objective moral truth any preliminary form of an identical and mass-employed ’moral calculator’ or ’advisor’ will be at least limited by even our current best conception of a comprehensive moral framework.

Consequently, continued reliance on automated aid in moral deliberation poses the risk that, to tackle higher-order problems, we may unintentionally and unnoticeably narrow our conception of morals by no longer challenging our more fundamental moral concepts. Thus, it seems the concept of supporting ’moral consistency’ is intertwined with the concept of ’moral efficiency’: We would choose to automate deliberation about lower-order moral dilemmas to make space for indulging in higher-order ones. To borrow an example from Giubilini and Savulescu (2018), we may disregard a moral duty to inform ourselves properly about particular forms of waste, potentially changing our habit of omitting it altogether, such as disposable coffee cups, but, instead, let the ’moral advisor’ steer our way to the most ecological disposal. Hence, automating moral deliberation by AMAs will almost certainly be incentivized by increasing productivity, which puts human scrutiny at risk, even considering AMAs in purely personal use.

Cave et al. (2018), argue that some moral duties may always remain irreconcilable—an idea put forward by pluralistic ethical frameworks, cf. (Mason 2018). Cave et al. illustrate that ’moral imperialism’ accrues when globally implementing an AMA that may implicitly prefer the values of the developing team. I think this is a valid objection. However, I would even maintain that premature forms of AMAs will always act in ways that are biased by the moral competency of development teams, or their strength of will to ’get it right’ despite economic interests and pressures. It is unlikely that the one product that best implements the current state-of-the-art of moral reasoning emerges from the market as most viable. Rather, the one that promises its consumers the most economic success will prevail—unless the ways our economy works changes drastically. It may well be objected that this is an issue with about any product. It may often be the case that products succeeding on the market are not the ones of highest quality, let alone the most ethical, but those that promise the greatest utility to its customer, financial, or otherwise. While I am not saying that this should be accepted, it surely may be even more significant with AMAs. This is because (i) AMAs may not only be less ethical, but in affecting a customer’s morals they may have an even potentially more significant impact on more, if not all, other areas of life, and (ii) merely by virtue of constituting systems concerned with morals, they may be perceived as carrying the acknowledged ’stamp’ of morality, reducing skepticism and scrutiny.

I will not long dwell on the subject of malicious use or interference with tools for moral deliberation, but—quite obviously—if no objective moral standard exists, developing companies could easily try and skew the outputs to their advantage, or anyone else’s for that matter. Alternatively, just like actuaries pressured by clients to arrive at decisions that are most profitable in the clients’ own short-term interests, developing companies can be pressured to supply AMAs programmed to favor the client. This is a link and also a valid point with the ’moral replicas’ from Sect. 3. To be viable on the market, manufacturers of AMAs would strive to create devices that realize a maximum of economic profitability for their customers. The developers’ task would be first to determine the red lines that may not be crossed, which in itself is first and foremost a legal exercise, and then devise an AMA that seeks the pareto-optimal decisions closest, but not surpassing, the red line constituting the threshold to illegality. Within this simple image, morality, however, would often rather demand finding the optimal decision farthest away from all red lines, something which is beyond the law to enforce. Thus, within this setting of AMAs as a service that promises economic benefits, what is likely to ensue is a race to the ethical bottom, by only satisfying only minimal moral requirements. This is the opposite of Machine Ethics’ noble goal to improve human morality.

Above, I have already suggested that regulations and codes of ethics may be put into place to alleviate the risk of AMAs being biased intentionally or accidentally. However, this may even be be counterproductive to the proclaimed goal of improving upon human morals and moral deliberation: To hold developing companies to account would very likely require standardization that, in itself, may contradict the current status quo of the discourse in ethics. By standardizing which moral framework to implement in AMAs, humankind could, de facto, limit its conception of moral deliberation within a narrow and potentially overly technocratic framework. Humankind could thus claim to have successfully constructed an AMA by adhering to the standard, but—by over-relying on it—it would have given up its pursuit of understanding the full extent of what moral agency constitutes and—even more severe—what needs to be included in any moral deliberation and why. This technocratic approach is also at the heart of the criticism raised by Gordon (2020).

### Disregard of an Agent’s Moral Interior

To support the point that AMAs provide the risk of a narrowing of human moral thought further, in the following, I will address the risk that AMAs may advance a disregard for the view that a moral agent’s interior is relevant for moral agency. Consider that recent research on the role of intuition and emotion in moral deliberation seems to support Aristotle’s concept of habituation (NE II.1, 1103a25-26) as the notion that repeated and recognized practice of virtuous actions is necessary to acquire practical wisdom (phronesis) and a virtuous character (Kraut 2018). Sauer (2012), e.g., argues that moral intuition is formed by prior rational deliberative processes. In other words: Rational reasoning may turn into increasingly automatic moral judgments. While current machine learning methods may be argued to be capable of mimicking the process of turning moral deliberation results into intuition, cf. (Yu et al. 2018), they are far from emulating any antecedent deliberation process. Thus, apart from the threat of mass automation of moral decision-making, devices such as ’moral calculators’ may not entirely prevent the formation of moral intuitions and lead to a complete moral deskilling, cf. (Vallor 2015). Instead, if over-relied upon, they may shape our intuitions in uniform ways, aligning them with the outputs of mass-replicated, premature, and deficient pieces of software.

To be concise, for this effect to take shape, I suggest that it requires the following ingredients:

(i) AMAs will be made available at mass-scale, such that uniform software solutions prevail over highly diverse and individual moral reasoning aids,
(ii) AMAs will be employed incentivized by increasing productivity, or personal ’moral efficiency,’ such that scrutiny is reduced, and
(iii) the use of AMAs for moral decision-making will crowd out human discourse over (daily) moral dilemmas, such that a lack of arguments fails to provide for corrective action on our moral intuitions, which are, hence, predominantly shaped by AMAs.

From the thought experiments I have elucidated above, I think it has become clear that the line of reasoning from items (i) and (ii) is plausible. Indeed, current advocates of AI systems for decision-support promise standardized systems for productivity increases, cf. (IBM 2019; Strickland 2019). Whether or not human interaction in discourse will reduce over the use of AMAs remains speculation.

Humankind may continue to (frequently) refer to other humans for moral council due to the notion of trust in character and, hence, a regard for the interior of other moral agents. Revealing intentions, feelings, and weaknesses to other human beings constitute an essential part of building trust. This also involves knowing a person’s reasons for his or her moral actions and perhaps evolution of the moral convictions, cf. (Kukita 2015).

However, at least in reference to AMAs, a closer look at some machine ethics literature reveals that the interior of a moral agent is disregarded and, already now, when there is only a distant prospect of implementing an AMA, it confines itself—in argument — to specific limited ideas of moral agency. Here, I am criticizing machine ethicists that confine their definitions of artificial moral agency to solely rely on assessing an AMA’s outputs based on what is deemed ’correct’ within the moral domain, such as Beavers (2011); Cave et al. (2018) (see a discussion of their definitions above). This includes advocates of a so-called Moral Turing Test (MTT), which is essentially satisfied when presenting an illusion of moral behavior, see, e.g. (Allen et al. 2000; Beavers 2012; Gerdes and Ohrstrom 2013; Arnold and Scheutz 2016). Proponents of the MTT may object that humans can also only assess other humans based on observed actions and statements. However, often our final verdict will be made in terms of notions of character that also aim at incorporating intentions, emotional states, and similar items we attribute to the interior of a moral agent. These more abstract concepts allow to extrapolate to moral qualities to be expected in future encounters. Incorporating a notion of ’character’ within AMAs thus appears much more likely to safeguard against potentially severe moral decisions in uncharted terrain. This surely is not a new suggestion and will likely not constitute a full solution to the issues and dangers, I have outlined so far. Rather, I would like to point out that it seems inconsistent to desire perfect safety by ethical alignment of artificial moral agents while only be interested in an input-output systems perspective for evaluating their moral agency, particularly since AMAs will be entities that are, presumably, entirely human-made.

Strong certification processes for initial versions of AMAs and regulations for employing these are certainly needed for ’ethical impact agents’ and those bordering onto ’implicit ethical agents.’ Highly relevant pioneering work is being conducted, e.g., by the IEEE with the introduction of the P7000 standards, see (Adamson et al. 2019). These standards attempt to devise norms on processes for designing and behaviors of autonomous systems concering, e.g., personalized AI systems (WG-PDAI - Personal Data AI Agent Working Group 2017). It is beyond the scope of this article to argue conclusively for the potential content of regulations. However, drawing up comprehensive and workable policies appears at least highly intricate, recognizing the global difficulties with tackling climate change through regulations, market-based incentives, etc. With AMAs, however, we may at least agree on a moratorium until citizens, politics, and science together have drawn up a working set of regulations.

Meanwhile, if the science behind constructing AMAs itself gives up on investigating the full extent of what constitutes moral deliberation, the utilization of AMAs to take over or aid in moral deliberation will not really assist humankind in making moral decisions. Rather, it will form what it means to make moral decisions. If these AMAs will turn out deficient and premature, they will form our conception of morals to include considerably less in terms of moral frameworks about character, intentions, or other aspects interior to moral agents. I believe that it is nothing less that is at stake here.

## Conclusion

In this article, I have argued against a premature practical and economical utilization of intermediate steps in the scientific pursuit to build AMAs. My contribution rests on stepping beyond statements about what AMAs may eventually end up to be from the perspective of an idealized linear development, but rather I have tried to sketch more imminent risks, supported by current developments, that point towards adverse short- to medium-term effects on our social order and the scientific pursuit towards higher human morality. In doing so, I have argued that preliminary forms of AMAs are likely to skew moral decisions towards moral norms determined by other factors such as implementation constraints or economic viability. Widespread use of initial and deficient forms of AMAs as aids for moral deliberation may shape our moral intuitions in ways limited by the premature state-of-the-art in AMA research. This may create a lock-in effect that may ultimately and prematurely end humankind’s pursuit of advancing towards a better understanding of what constitutes moral truth and the workings of human moral thought. This is because we have altered and effectively limited the subject that we set out to investigate scientifically to begin with. In addition, I have also argued that within a socio-technical context, premature employment of AMAs poses the risk of consolidating or increasing power asymmetries within society. Therefore, at least in the short term, we should proceed very cautiously and engage in inclusive discourse about potential regulations for allowing practical use of premature forms of AMAs, if we are to allow this at all.

## Data Availability

Not applicable.
